# Glomerular hyperfiltration and β-2 microglobulin as biomarkers of incipient renal dysfunction in cancer survivors

**DOI:** 10.4155/fsoa-2018-0045

**Published:** 2018-08-10

**Authors:** Fernanda R Tibúrcio, Karla E de S Rodrigues, André R Belisário, Ana Cristina Simões-e-Silva

**Affiliations:** 1 Department of Pediatrics, Interdisciplinary Laboratory of Medical Investigation, Faculty of Medicine, Pediatric Nephrology Unity, Federal University of Minas Gerais (UFMG), Belo Horizonte, Brazil

**Keywords:** β-2 microglobulin, glomerular hyperfiltration, nephrotoxicity, pediatric cancer, subclinical renal dysfunction

## Abstract

Herein, we aimed to evaluate the occurrence of impaired renal function after cancer treatment with potentially nephrotoxic chemotherapy in children. A cross-sectional study was performed in 41 cancer survivors after chemotherapy with potentially nephrotoxic drugs. 26 (63.4%) children were detected with glomerular hyperfiltration, and urinary levels of β-2 microglobulin (B2MG) were higher than reference range in all patients. Levels of B2MG were positively correlated with plasma creatinine and negatively correlated with glomerular filtration rate. Plasma creatinine, systolic blood pressure and cholesterol were independently associated with B2MG values. The final multivariate model for glomerular hyperfiltration risk included plasma levels of urea and of magnesium. Urinary levels of B2MG and glomerular hyperfiltration may emerge as potential biomarkers of early renal dysfunction in childhood cancer survivors.

## Lay abstract

Herein, we evaluated the occurrence of renal dysfunction following cancer treatment in children. This study included 41 cancer survivors treated with medications that may damage the kidney. 26 children exhibited glomerular hyperfiltration, and urinary levels of β-2 microglobulin (B2MG; a protein linked with tubular dysfunction) were high in all patients. Plasma creatinine, blood pressure and cholesterol levels were associated with B2MG. Plasma levels of urea and of magnesium were associated with glomerular hyperfiltration. Urinary levels of B2MG and glomerular hyperfiltration may emerge as potential biomarkers of early renal dysfunction in childhood cancer survivors.

Over the last few decades, substantial advances in cancer management have decreased mortality [1]. However, chemotherapy commonly used in the treatment of cancers may cause nephrotoxicity [1,2]. Nephrotoxicity induced by chemotherapy occurs with variable frequency in cancer survivors. There are several studies on nephrotoxicity induced by chemotherapy in adult patients. However, the real prevalence and severity in childhood cancer survivors remain uncertain, mainly due to differences in studied groups, treatment and methods to evaluate renal toxicity [2,3]. Renal toxicity may manifest as reversible acute kidney injury, but with potential to cause chronic kidney disease (CKD) [2,3]. Nephrotoxicity may lead to renal abnormalities, resulting in glomerular and/or tubular dysfunction. The evaluation of nephrotoxicity has been frequently based only on the determination of plasma urea and creatinine concentrations, as well as urinary protein excretion [1,2]. However, subclinical renal dysfunction is not uncommon in cancer survivors, and these previously mentioned tests are not able to detect early changes in kidney function [4]. Therefore, potential biomarkers of early renal injury should be evaluated in cancer survivors, mostly in children.

Some studies have proposed new biomarkers for early detection of renal damage, including kidney injury molecule, IL-18, and β-2 microglobulin (B2MG) [5–7]. However, most of these potential biomarkers have not always been associated with a renal disease outcome. Recently, in a single center study, B2MG and IL-18, but not kidney injury molecule, were associated with CKD in children after chemotherapy [7]. Moreover, only a few studies have evaluated potential biomarkers of kidney function after chemotherapy in children. In this context, our study aimed to investigate the occurrence of impaired renal function after cancer treatment with potentially nephrotoxic chemotherapy in children from a single pediatric oncology unit by adopting a systematic protocol to assess early glomerular and tubular alterations.

## Methods

### Study design & patients

This was a cross-sectional study conducted between February 2013 and July 2014. Study subjects comprised 41 recruited children, who were previously diagnosed with a solid malignancy and had already completed cancer treatment for at least 1 month and were in remission. Children were followed-up at Pediatric Oncology unit. The patients received at least one of the following potentially nephrotoxic chemotherapy drug: cisplatin, carboplatin, ifosfamide, cyclophosphamide and methotrexate. No selected children presented acute kidney injury during treatment.

The study was approved by institutional review board (reference number 346/13). Informed written consents were obtained from participants’ parents or guardians and the children's assents were also obtained, when appropriate. The study was conducted in accordance with the Declaration of Helsinki guidelines.

### Clinical & laboratory parameters

Chart review for each patient was performed to collect data about cancer treatment. All patients underwent complete physical examination by a single physician. Blood pressure was measured with the standard method. Systolic and diastolic blood pressure percentiles were evaluated according to age, gender and height of the patients [8]. Nutritional status was evaluated using criteria based on WHO child growth standards [9]. Body mass index (BMI) Z scores and percentiles were classified using previously proposed charts [10].

Two urine samples were collected from each child. One sample was used to carry out urinalysis. The second sample was divided in two vials: the first one was to assess urine pH and osmolality; the second one was to quantify microalbuminuria (turbidimetric method, Quibasa/Bioclin K078 kit, Belo Horizonte, MG, Brazil) and B2MG (ELISA, R&D Systems Kit, MN, USA). Immunoassays (ELISA) were read at a wavelength of 450 nm using a microplate reader (Tp Reader, ThermoPlate, OH, USA). Following the manufacturer's instructions, the cut-off points were 0.3 μg/ml and 30 μg/mg for B2MG and microalbuminuria, respectively. Urine pH was measured just after urinary collection in a pH meter (pH-Meter HI 4222-2, Hanna Instruments Deutschland GmbH, Vöhringen, Deutschland) and urine osmolality was determined by a freezing-point osmometer (microOsmetter, IL, USA).

A 24-h urine collection was performed to measure creatinine and electrolytes. Creatinine was measured by Isotope dilution mass spectrometry (IDMS) traceable method. Calcium, phosphorus and magnesium were assayed by colorimetric method, whereas sodium, potassium by ion-selective electrodes, and chloride by potentiometric titration.

We also collected a blood sample for measurements of urea, creatinine, sodium, potassium, calcium, phosphorus, magnesium, chloride, venous blood gas, fasting blood glucose, total cholesterol and fractions, triglycerides and total protein and fractions. Patients were fasted for 8–12 h before blood collection. Blood creatinine and electrolytes were quantified using the same methods mentioned above. Urea, total cholesterol and fractions, and total protein and fractions were assayed by colorimetric method. Triglycerides and fasting blood glucose were determined using an enzymatic-colorimetric assay and enzymatic assay, respectively. Venous blood gas was measured by blood gas analyses (Blood Gas Analyzer, Medica, MA, USA). Urine and blood samples were collected during the follow-up visits after the end of cancer treatment. The median follow-up period was 3.5 years, ranging from 2 months to 13 years.

### Outcomes

Glomerular filtration rate (GFR) was calculated according to Schwartz modified formula [11]. Altered glomerular function was defined as GFR lower than 90 ml/min/1.73 m^2^ and/or GFR higher than 175 ml/min/1.73 m^2^ (glomerular hyperfiltration) [12] and/or microalbuminuria higher than 30 mg/g creatinine. Microalbuminuria was defined by urine albumin/creatinine ratio (UACR) 30–300 mg/g creatinine. Proteinuria was defined by albumin/creatinine greater than 300 mg/g creatinine. Altered tubular function was defined as the presence of glycosuria, metabolic acidosis and/or abnormal electrolytes loss in the urine. ‘Nephrotoxicity’ was defined in the present study by the occurrence of altered glomerular and/or tubular function in the absence of other causes.

### Statistical analysis

Median, mean and standard deviations (SD) were calculated for continuous variables and categorical variables were expressed as percentages. The Shapiro–Wilk test was used to evaluate the normal distribution of continuous variables. The unpaired *t*-test and nonparametric Mann–Whitney U test were used to compare different groups for continuous variables that were normally and not normally distributed, respectively. Levene's test was used to assess the homogeneity of variances. Univariate association between categorical variables was evaluated using the two-tailed chi-square or Fisher's exact test. Pearson or Spearman correlation coefficient was used to assess correlation between continuous variables.

B2MG values were higher than reference values in all children. Then, multivariate linear regression was performed to identify covariates independently associated with B2MG values. Significantly associated variables in a univariate analysis (p < 0.05) were assessed simultaneously in a multiple linear regression model using backward elimination. The significance level was defined as two-sided, p < 0.05 for all analysis.

Cox's regression was used to determine the independent effect of each covariate associated with ‘glomerular hyperfiltration’. The initial multivariate model included all covariates potentially associated with outcome (p < 0.20 in univariate analysis). The covariates with higher p-value were removed from the model through backward elimination and the final model included only those covariates that were statistically significant at p < 0.05. Statistical analyses were performed with SPSS 19.0 software (SPSS Inc., IL, USA).

## Results

### Patient characteristics

41 pediatric patients were included in the study; 21 (51.2%) were male. The median age at cancer diagnosis was 9 (range: 8 m–15 y). The median follow-up period was 3.5 years (range: 2 m–13 y). The etiologies of tumors were: six (14.6%) nasopharyngeal carcinoma, six (14.6%) Ewing's sarcoma/primitive neuroectodermal tumor, six (14.6%) germ cell tumor, five (12.2%) osteosarcoma, four (9.8%) medulloblastoma, three (7.3%) neuroblastoma, two (4.9%) soft tissue sarcoma, one (2.4%) retinoblastoma, one (2.4%) astrocytoma and one (2.4%) hepatoblastoma. Eight patients (19.5%) had metastatic stage IV, 31 (75.6%) stage III and 2 (4.9%) presented with stage II. 20 (48.8%) children received cisplatin, 17 (41.8%) received cyclophosphamide, 15 (36.6%) ifosfamide, nine (22.0%) carboplatin and four (9.8%) methotrexate. With regard to concomitant use of these potentially nephrotoxic drugs: 11 (26.8%) children received only cisplatin; five (12.2%) received ifosfamide and cyclophosphamide; four (9.8%) only carboplatin; four (9.8%) only cyclophosphamide; four (9.8%) cisplatin and methotrexate; four (9.8%) ifosfamide, carboplatin and cyclophosphamide; three (7.3%) only ifosfamide; three (7.3%) carboplatin and cyclophosphamide; two (4.9%) ifosfamide and carboplatin; and one (2.4%) child received ifosfamide and cisplatin. No patient had weight-for-age, weight-for-height and height-for-age below -2 Z-scores. One (2.4%) child hada BMI above the 85th percentile. No patient had hypertension. Clinical data of the patients were summarized in Table 1.

**Table 1.** **Clinical and laboratory data of 41 childhood cancer survivors post-treatment.**

**Variable**	**Mean (standard deviation)**	**Minimum**	**Maximum**	**Shapiro–Wilk test p-value**
Weight (kg)	51.2 (17.1)	16.0	79.0	0.618

Height (cm)	151.3 (20.3)	95.0	182.0	0.088

Body mass index	26.0 (4.6)	19.7	32.3	0.105

Systolic BP (mmHg)	106.3 (12.4)	80.0	130.0	0.008^†^

Diastolic BP (mmHg)	68.2 (10.5)	40.0	80.0	0.061

Systolic BP percentile	–	15	85	–

Diastolic BP percentile	–	10	80	–

Urea (mg/dl)	26.8 (7.3)	12.0	41.0	0.942

Creatinine (mg/dl)	0.69 (0.23)	0.30	1.64	0.539

Cholesterol (mg/dl)	177.2 (21.6)	135.0	212.0	0.904

Blood glucose (mg/dl)	81.5 (5.2)	70.0	92.0	0.403

Triglycerides (mg/dl)	109.6 (40.6)	58.0	264.0	0.170

Protein (g/dl)	7.9 (0.3)	7.3	8.4	0.406

Na (mEq/l)	138.9 (2.0)	134.0	142.0	0.254

K (mEq/l)	4.1 (0.2)	3.5	4.7	0.311

Ca (mg/dl)	9.9 (0.4)	9.0	10.7	0.599

Mg (mg/dl)	1.9 (0.2)	1.5	2.4	0.499

P (mg/dl)	4.7 (0.6)	3.3	5.8	0.985

Cl (mmol/l)	103 (1.8)	99	107	0.882

BIC (mmol/l)	25.2 (1.7)	20.6	29.4	0.789

Urine pH	5.7 (0.3)	4.8	6.0	0.022^‡^

OSM (mOsmol/kgH_2_O)	628.0 (150.0)	286.0	887.0	0.553

Microalbuminuria (mcg/mg creatinine)	7.6 (4.5)	0.1	22.0	0.457

β2MG (μg/ml)	12.1 (5.5)	2.7	27.6	0.986

24-h urine volume (ml)	1533.8 (382.4)	875.0	2.200.0	0.570

Urine creatinine (mg/dl)	66.2 (29.8)	12.7	194	0.539

24 h urine creatinine (mg/24 h)	618.1 (297.7)	0	1365	0.907

Estimated GFR (ml/min/1.73 m^2^)	187.7 (50.7)	78	338	0.222

Fractional excretion of Na (%)	0.6 (0.2)	0.1	0.9	0.291

Fractional excretion of P (%)	12.0 (3.4)	4.0	18.0	0.670

Fractional excretion of Ca (%)	0.8 (0.3)	0.4	1.2	0.006^†^

Fractional excretion of Mg (%)	1.2 (0.2)	0.6	1.8	0.897

Fractional excretion of K (%)	15 (2.9)	8	22	0.803

^†^Significant p-values (variables not normally distributed).

^‡^Significant p-values (variables with normal distribution).

B2MG: β-2 microglobulin; BIC: Bicarbonate; BP: Blood pressure; Ca: Calcium; Cl: Chloride; GFR: Glomerular filtration rate; K: Potassium; Mg: Magnesium; Na: Sodium; P: Phosphate.

### Laboratory data

Plasma levels of urea were within the reference range for all patients. One child had plasma creatinine greater than age-specific reference range (1.64 mg/dl). Blood glucose, total protein and plasma albumin values were within the normal range for all patients. Electrolytes and fractional excretion of electrolytes were also within the normal range for all patients. 27 (65.8%) children had cholesterol levels above 170 mg/dl and five (12.2%) had cholesterol levels above 200 mg/dl. Nine (21.9%) children had triglyceride levels higher than 130 mg/dl. The blood pH and CO_2_ partial pressure were within the reference range for all children. Consequently, plasma bicarbonate concentration was also normal. B2MG values were higher than reference range for all patients.

The urine pH and osmolality were within the normal range for all patients. 24 h urine volume was considered normal for all children. There was no estimated GFR lower than 60 ml/min/1.73 m^2^. One (2.4%) child had a slightly decreased estimated GFR (78 ml/min/1.73 m^2^); the same child had increased levels of plasma creatinine (1.64 mg/dl). However, 26 (63.4%) patients presented glomerular hyperfiltration (GFR ≥ 175 ml/min/1.73 m^2^). No child had a UACR greater than 30 mg/g or proteinuria. According to the definition used in this study, 23 (63.4%) children presented ‘nephrotoxicity’ (all of them due to glomerular hyperfiltration). Laboratory data of the patients were summarized in Table 1.

### Correlation analysis between B2MG & continuous variables

**Figure 1.**
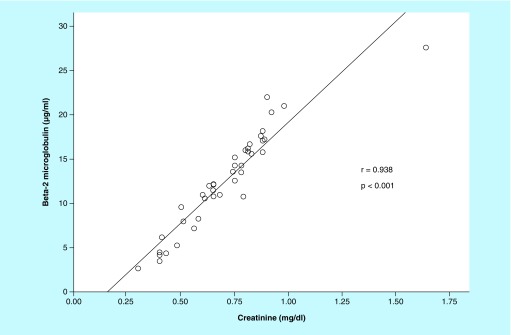
**Correlation between urinary levels of β2-microglobulin and plasma levels of creatinine in 41 pediatric cancer survivors post-treatment.**

There was a strong positive correlation between B2MG and plasma levels of creatinine (r = 0.938; p < 0.001; Figure 1). Accordingly, there was a moderate negative correlation between B2MG values and estimated GFR (r = -0.611; p < 0.001). There was a moderate positive correlation between B2MG values and systolic blood pressure, height, weight and 24-h urine volume (0.4 ≤ r ≤ 0.7; p < 0.05). There was a weak positive correlation between B2MG and plasma levels of cholesterol, urea and magnesium (r < 0.4 and p < 0.05).

### Association between B2MG & categorical variables

There was no statistically significant association between B2MG values and gender, presence of metastasis, metastasis stage, type of chemotherapy, BMI percentiles and blood pressure percentiles (data not shown; p > 0.05).

### Impact of concomitant covariates on the B2MG value: multivariate linear regression analysis

We also studied the clinical and laboratory features that were independently associated with B2MG values. We included all plausible biological covariates in the initial model using a multivariable linear regression (Table 2). We subsequently removed the covariates from the model, one by one through backward analysis and the final model included only those covariates with p-values < 0.05. In the final model, total cholesterol, plasma levels of creatinine and systolic blood pressure were significantly associated with B2MG values (Table 2). This final multivariate model explains 92.4% of the variability in B2MG values. The most significant covariate of the final model was plasma creatinine. We performed multicollinearity tests based on the variance inflation factor, which indicated the absence of multicollinearity problems (Table 2).

**Table 2.** **Multivariable linear regression models of variables independently associated with urinary levels of β-2 microglobulin among 41 childhood cancer survivors post-treatment.**

**Variable**	**B**	**β**	**p-value**	**Partial R**	**T**	**VIF**
**Initial model: all covariates**						

Constant	-20,075	–	0.001	–	-3,877	–

Ifosfamide use (yes/no)	-0,858	-0,076	0.304	-0,201	-1,048	2,213

Cisplatin use (yes/no)	-1,079	-0,099	0.306	-0,2	-1,043	3,802

Cyclophosphamide use (yes/no)	-0,082	-0,007	0.903	-0,024	-0,123	1,552

Carboplatin use (yes/no)	-0,411	-0,031	0.645	-0,091	-0,466	1,893

Methotrexate (yes/no)	-0,098	-0,005	0.936	-0,016	-0,081	1,822

Follow-up (years)	-0,004	-0,035	0.666	-0,085	-0,437	2,777

Age at diagnosis (years)	-0,059	-0,047	0.583	-0,108	-0,556	3,004

Metastasis (yes/no)	0,043	0,003	0.96	0,01	0,051	1,573

Gender (male/female)	-0,494	-0,045	0.477	-0,14	-0,722	1,667

Plasma levels of magnesium (mg/dl)	1,811	0,059	0.394	0,168	0,867	1,968

Plasma levels of urea (mg/dl)	-0,063	-0,084	0.296	-0,205	-1,067	2,604

Plasma levels of cholesterol (mg/dl)	0,042	0,166	0.031	0,408	2,282	2,224

Plasma levels of creatinine (mg/dl)	19,878	0,821	< 0.001	0,919	11,91	2,003

Systolic blood presure (mmHg)	0,104	0,233	0.009	0,487	2,845	2,829

**Final model: only covariates with p < 0.05**						

Constant	-15,472	–	< 0.001	–	-5,725	–

Plasma levels of cholesterol (mg/dl)	0,036	0,142	0.005	0,439	2,975	1,107

Plasma levels of creatinine (mg/dl)	19,784	0,817	< 0.001	0,926	14,968	1,451

Systolic blood presure (mmHg)	0,07	0,158	0.007	0,425	2,854	1,493

B: Unstandardized coefficient; β: Standardized coefficient; OSM: Osmolality; Partial R: Partial correlation coefficient; T: *t*-statistic; VIF: Variance inflation factor.

### Univariate analysis of variables associated with glomerular hyperfiltration

Univariate analysis identified four factors that significantly associated with glomerular hyperfiltration (p < 0.05). Continuous variables serum levels of Na (mEq/l), of Mg (mg/dl), urinary osmolality (mOsmol/kg H_2_O) and urinary levels of B2MG (μg/ml) were significantly lower in the group of patients with hyperfiltration when compared with those without hyperfiltration. There was no statistically significant association between hyperfiltration and gender, presence of metastasis, metastasis stage, type of chemotherapy, BMI percentiles and blood pressure percentiles (data not shown; p > 0.05).

### Multivariate analysis of variables independently associated with glomerular hyperfiltration

The final multivariate model for variables independently associated with glomerular hyperfiltration included serum levels of urea (mg/dl) and of magnesium (mg/dl) (Table 3). The most significant covariate of the final model was serum levels of magnesium.

**Table 3.** **Multivariable Cox's regression model of variables independently associated with glomerular hyperfiltration among 41 childhood cancer survivors post-treatment.**

**Covariate**	**Hazard ratio**	**95% CI**		**p-value**

		**Lower**	**Upper**	
**Initial model: covariates with p < 0.20 in univariate analysis**				

**Metastatic stage**				

Stage II	Reference			

Stage III	5.722	0.346	94.737	0.223

Stage IV	1.170	0.336	4.073	0.805

Systolic blood pressure	1.009	0.933	1,090	0.830

Urine pH	0.930	0.168	5.155	0.934

Plasma levels of urea	0.888	0.821	0.959	0.003

Plasma levels of sodium	1.099	0.816	1.480	0.535

Plasma levels of calcium	0.284	0.078	1.039	0.057

Plasma levels of magnesium	0.019	0.001	0.516	0.019

Urinary osmolality	0.998	0.995	1.002	0.387

Urinary levels of β2MG	0.942	0.774	1.145	0.547

**Final model: covariates with p < 0.05 after backward elimination**				

Plasma levels of urea	0.902	0.846	0.962	0.002

Plasma levels of magnesium	0.042	0.003	0.550	0.016

β2MG: β-2 microglobulin; CI: Confidence interval.

## Discussion

In this study, we detected 63.4% of glomerular hyperfiltration and high levels of B2MG in all patients. B2MG values were positively correlated with plasma creatinine values and negatively correlated with estimated GFR. We also examined variables associated with urinary levels of B2MG and with glomerular hyperfiltration in childhood cancer survivors post-chemotherapy.

Glomerular dysfunction may occasionally cause abnormally elevated plasma urea and creatinine levels, suggesting transitory or persistent glomerular injury. However, such changes only appear in a minority of patients [2,4,13,14]. How to recognize early stages of post-chemotherapy nephrotoxicity remains a challenge. Creatinine and urea levels monitoring are certainly not sufficient, because increased plasma levels occur only in advanced stages of renal injury. Accordingly, in our study, only one child showed high levels of plasma creatinine and none showed abnormal levels of urea. This finding is in agreement with those of other investigators. Changes in creatinine levels were also not observed in 18 patients treated with a regimen using cisplatin [15]. Small increases in creatinine levels have been associated with clinical morbidity. However, rise in creatinine level is a late marker, usually observed with irreversible renal damage [2,16].

In this study, only one (2.4%) child had a slightly decreased estimated GFR. Previous reports showed that reduction in GFR is relatively common after chemotherapy, affecting about 50% of patients [17]. However, in our study, patients treated with radiotherapy and with nephrectomy were excluded and this fact may provide a plausible explanation for lower prevalence of reduction in GFR. In contrast, glomerular hyperfiltration was a very common finding in this study. Glomerular hyperfiltration has been associated with early stages of nephropathy that evolved to CKD [18]. Glomerular hyperfiltration has also been linked with obesity, metabolic syndrome [19] and early stages of sickle cell nephropathy [20].

Frequency of glomerular hyperfiltration is higher in cancer survivors after treatment with chemotherapy [21] than in children with cancer at diagnosis [12]. A previous study reported glomerular hyperfiltration in 58.8% of pediatric survivors of acute lymphoblastic leukemia following chemotherapy and bone marrow transplantation [21]. A more recent study has reported glomerular hyperfiltration in 31% children with cancer at diagnosis, before chemotherapy [12]. The mechanisms responsible for glomerular hyperfiltration in patients with cancer are not fully understood, but certainly involve factors related with the disease itself and secondary effects of chemotherapy [12,22]. As hyperfiltration may result from inflammatory and oxidative aggression to the kidney, we hypothesized that chemotherapy-induced nephrotoxicity could also induce glomerular hyperfiltration by similar mechanisms. Experimental studies are needed to confirm this hypothesis.

To the best of our knowledge, this is the first study to evaluate factors independently associated with glomerular hyperfiltration in patients with cancer. The multiple regression Cox's model showed a significant and independent association between plasma levels of urea and glomerular hyperfiltration. The chance of glomerular hyperfiltration decreased by 0.9 per each unit increase in plasma levels of urea. The precise meaning of this association is not very clear. Since urea levels were measured after the use of nephrotoxic chemotherapy, it can be speculated that low levels of urea in plasma are a consequence, rather than a cause, of glomerular hyperfiltration. Plasma levels of magnesium were also significantly and independently associated with glomerular hyperfiltration in both univariate and multivariate analyses. Reductions in plasma levels of magnesium increased the chance of glomerular hyperfiltration. The explanation for this finding is also intriguing. Indeed, reduction in plasma levels of magnesium may reflect mild alterations in distal tubule mechanisms of reabsorption of this ion. In addition, low magnesium level has been associated with endothelial dysfunction, and consequently, oxidative damage and inflammation in renal tissue [23]. These mechanisms may, at least in part, contribute to glomerular hyperfiltration.

In this study, childhood cancer survivors did not show UACR greater than 30 mg/g post-treatment. A previously published study showed that 30% of children after anticancer treatment presented increased UACR levels [2]. The most likely explanation for the lack of UACR greater than 30 mg/g in our study was the relatively short follow-up period.

Over the past few years, B2MG has been evaluated as a biomarker of kidney injury [24]. In this study, we found high urinary levels of B2MG in all children. This finding is in agreement with Sorensen *et al*. [15] that reported a two- to fivefold increase in B2MG excretion in the urine of all 18 patients after cisplatinum treatment. In a study with 85 children, urinary levels of B2MG were also significantly higher after chemotherapy [7]. B2MG has also been considered a marker of tubular dysfunction [25]. Some authors also suggest the use of B2MG to monitor kidney function in other conditions, such as Type 2 diabetes [26]. Tubular injury may precede or co-exist with glomerular injury [27]. In this regard, we found a negative correlation between urinary levels of B2MG and GFR calculated by modified Schwartz formula [11]. This correlation may indicate subclinical kidney disease. However, one previous study did not find correlation between B2MG levels and GFR [7]. Another report showed that the decrease in creatinine clearance was not correlated to either the peak increase in the B2MG excretion or to the time of occurrence of the peak [15]. Furthermore, Meijer *et al*. [28] showed that reduction in GFR occurs without change in B2MG levels during treatment with cisplatin. However, these previous studies analyzed B2MG levels during chemotherapy and not after the treatment as in our study. Thus, comparisons are not very reliable.

More recently, B2MG concentrations have been reported as elevated at the time of diagnosis in many solid and hematological cancer and may serve as a marker of early detection for disease relapse [29]. Pretreatment levels of B2MG may also be considered a prognostic factor in Hodgkin lymphoma [30] and in diffuse large B-cell lymphoma [31]. However, it is important to mention that in our study all patients were enrolled after the end of the treatment with disease in remission. Our patients had undergone physical and image examination to exclude active disease. Therefore, these studies of hematological cancers were different from our study in regard to the time of B2MG evaluation.

In this study, there was no apparent disorder of tubular function. All children showed normal ability to concentrate, to dilute and to acidify the urine. Most tubular nephrotoxicity in children with cancer occurs during treatment. Therefore, follow-up period relatively short was unlike to be the cause of the absence of renal tubular disorders [32]. A possible reason is that our patients were older than in previous studies [1–3]. Supporting this possibility is the observation that younger patients have significantly higher frequency of tubulopathies than older patients [33,34]. Furthermore, children treated with radiotherapy and with nephrectomy were included in the majority of previously reported studies, and this fact probably contributed to higher occurrence of nephrotoxicity.

This study has several limitations, mostly the small number of patients from a single center. Another issue is that children were followed-up for a relatively short period of time, thus precluding the evaluation of long-term renal complications. Confounding factors may also have been not recognized, including the use of other nephrotoxic drugs (antibiotics and antifungal) and clinical complications (infections, metabolic disorders, hypovolemia). Another limitation was the inability to evaluate the presence of pre-existing renal dysfunction. Furthermore, methods to estimate GFR are usually inaccurate, and tend to overestimate true GFR [22]. For this reason, we decided to adopt a high threshold definition of estimated GFR. However, it is possible that not all children with estimated GFR ≥ 175 ml/min/1.73 m^2^ have real glomerular hyperfiltration.

In summary, two-thirds of our patients exhibited glomerular hyperfiltration and all had high levels of B2MG in their urine. Since glomerular hyperfiltration has been associated with progressive nephropathy, children with cancer should be considered for renal disease risk monitoring. Further studies are required to establish whether urinary levels of B2MG and glomerular hyperfiltration are early predictors of long-term nephrotoxicity.

## Future perspective

Despite the fact that childhood cancer survival has been improved, long-term nephrotoxicity has been described as an important cause of decrease in health-related quality of life. For this reason, future studies addressing new effective therapeutic approaches with less nephrotoxic potential should be developed.

Meanwhile, laboratorial tests for the prediction of the risk for developing chronic renal disease and also for the early detection of incipient and still-reversible toxicity remain as useful tools.

**Summary points**

- Nephrotoxicity induced by chemotherapy occurs with variable frequency in cancer survivors.
- Prevalence and severity of nephrotoxicity induced by chemotherapy in childhood cancer survivors remain uncertain.
- Glomerular hyperfiltration was detected in 63.5% of cancer survivors.
- Urinary levels of β-2 microglobulin (B2MG) were higher than reference range in all cancer survivors.
- Plasma creatinine was strongly and positively correlated with urinary levels of B2MG.
- Plasma creatinine, systolic blood pressure and total cholesterol were independently associated with B2MG values.
- The final multivariate model for glomerular hyperfiltration risk included plasma levels of urea and of magnesium.
- Urinary levels of B2MG and glomerular hyperfiltration may emerge as early biomarkers of nephrotoxicity in pediatric cancer survivors.
